# Value, but high costs in post-deposition data curation

**DOI:** 10.1093/database/bav126

**Published:** 2016-02-09

**Authors:** Petra ten Hoopen, Clara Amid, Pier Luigi Buttigieg, Evangelos Pafilis, Panos Bravakos, Ana M. Cerdeño-Tárraga, Richard Gibson, Tim Kahlke, Aglaia Legaki, Kada Narayana Murthy, Gabriella Papastefanou, Emiliano Pereira, Marc Rossello, Ana Luisa Toribio, Guy Cochrane

**Affiliations:** 1 European Molecular Biology Laboratory, European Bioinformatics Institute (EMBL-EBI), Wellcome Genome Campus, Hinxton, Cambridge CB10 1SD, UK; 2 Alfred-Wegener-Institut Helmholtz-Zentrum für Polar-und Meeresforschung, Am Handelshafen 12, Bremerhaven 27570, Germany; 3 Institute of Marine Biology, Biotechnology and Aquaculture, Hellenic Centre for Marine Research, Heraklion, Crete, P.O. Box 2214 71003, Greece; 4 CSIRO Marine and Atmospheric Research, Castray Esplanade, Hobart TAS 7001, Australia; 5 Pondicherry University, Brookshabad Campus, Andaman and Nicobar Islands, Port Blair 744112, India; 6 Max Planck Institute for Marine Microbial Ecology, Microbial Genomics and Bioinformatics Group, Celsiusstr. 1, Bremen 28359, Germany

## Abstract

Discoverability of sequence data in primary data archives is proportional to the richness of contextual information associated with the data. Here, we describe an exercise in the improvement of contextual information surrounding sample records associated with metagenomics sequence reads available in the European Nucleotide Archive. We outline the annotation process and summarize findings of this effort aimed at increasing usability of publicly available environmental data. Furthermore, we emphasize the benefits of such an exercise and detail its costs. We conclude that such a third party annotation approach is expensive and has value as an element of curation, but should form only part of a more sustainable submitter-driven approach.

Database URL: http://www.ebi.ac.uk/ena

## Background

Annotation is a process in which contextual information is applied to data. Biological sense can only fully be derived from sequence data when accurate and adequate contextual information is available. This information is essential for data to be discoverable by the user community and to lead to deep interpretation. Despite this, sufficient contextual annotation of sequence data is frequently lacking in publicly available data sets. As a consequence, a data set lacking sufficient details on what was sampled, where, when and by whom it was sampled, and how it has been sequenced is not easily discoverable and if coincidentally discovered not usable due to low confidence in such data and difficulties to make comparisons to a seemingly similar data set.

To demonstrate the value of contextual data on one example, samples of the study PRJEB5982 were originally submitted without any sample attributes. Adding a single sample attribute specifying the geographic origin of the samples, in this case India, will allow users mining for sequence data from this geographic region to find data of the study PRJEB5982. Without this sample attribute using this search criterion data of the PRJEB5982 study would not be discovered.

The primary nucleotide sequence data archives, which host the world’s sequence data output, play two key roles in the integration, preservation and presentation of sequence data and related contextual information. First, these resources store, and make available for search and download, contextual information alongside sequence data. Second, these resources are in direct contact with molecular data providers and are therefore uniquely placed to capture, structure and integrate contextual data with sequence data at the time of data deposition.

The European Nucleotide Archive [ENA (1)], GenBank in the USA (2) and DDBJ in Japan (3) form the International Nucleotide Sequence Database Collaboration [INSDC (4)], a permanent and comprehensive repository for public domain nucleotide sequence data. Data and contextual information are exchanged between archives on a daily basis. This requires a high level of data harmonization among these repositories, which is implemented by supporting common data formats as well as contextual data standards developed by the INSDC and in collaboration with domain experts.

Contextual information is captured as sets of descriptors (in the form of key-value pairs) attached to a sample record, an abstraction representing the material to which sequencing has been applied (such as a sample of a microbial community or a plant tissue). ENA provides a growing number of checklists of sample descriptors (5) that facilitate contextual data reporting in compliance with the appropriate domain-specific data standards (6). For instance, microbial pathogen samples are described using descriptors from a pathogen-specific checklist while marine microbial samples use a marine-specific checklist.

## Post-deposition annotation exercise

In order to estimate the level of effort needed to improve the value and discoverability of molecular data postsubmission, we performed an annotation exercise on previously deposited records. We chose for our use case shotgun and amplicon metagenomics studies, where it is particularly important to report and record the environmental context information.

In the first stage of the exercise, we organized the Sample Record Annotation Workshop (SRAW), a 5-day intensive jamboree aimed to enrich contextual information in sample records, which were (i) openly available in the public domain, (ii) had been submitted into the ENA, (iii) were associated with metagenomic sequence data and (iv) were not available at the EMBL-EBI Metagenomics portal, a key provider of metagenomics analysis [EMG (7)], due to a lack of contextual data.

Sample records were annotated with contextual information mined from the literature and available in the public domain on the Web. Our goal was to enrich contextual information attached to the selected sample records and thus make these data sets more discoverable and meaningful. We approached this goal with two early and direct outputs in mind: First, the applied descriptors would be indexed in the ENA search service, allowing these records to become discoverable as users search the content for these types of data. Second, the improved sample records would become available for inclusion in EMG; here, we aimed to double the number of sample records available to this resource.

Six ENA staff curators were joined by eight invited, doctoral and postdoctoral-level researchers with backgrounds in biological sciences. The workshop hosted invited annotators from the HCMR Greece (8), MPI Bremen Germany (9), AWI Germany (10), CSIRO Australia (11) and Pondicherry University India (12). At the start of the SRAW, all participants were introduced to the ENA sample record concept (13) and relevant molecular data standards (6). A number of ontologies and ontology-related tools and services were also introduced. These included the Environment Ontology [ENVO, (14)], the Uber-anatomy Ontology [UBERON, (15), a *beta* version of the EXTRACT tool (16), ONTOBEE (17), BioPortal (18), OLVis (19) and the EBI Ontology Lookup Service (20)]. Additionally, procedures on requesting new ontology content via, e.g. the ENVO issue tracker (21) were introduced to encourage annotators to help shape the ontologies from which they drew. Supplementary controlled vocabularies such as the INSDC-Country vocabulary (22) were also introduced.

In order to help the annotators to (i) confidently use available tools, (ii) efficiently assess the sample records and (iii) extract relevant contextual information, an extensive preparation and pre-processing phase was necessary in advance of the SRAW. This consisted of (i) selecting suitable studies from ENA, (ii) generating a master annotation file with accessions and mappings, (iii) selecting, modifying and testing annotation tools, (iv) designing and documenting an annotation workflow and (v) preparing introductory training sessions relevant to the SRAW.

Annotators were provided with the documented workflow of the annotation process and used the master annotation file comprising (i) study record` accessions associated with metagenomic shotgun sequences absent in the EMG, (ii) sample record accessions corresponding to these environmental study accessions, (iii) existing contextual information descriptors (in the form of key-value pairs) corresponding to these sample records. Annotators began to work in three teams of four and as their annotation fluency increased, these teams were subdivided. The annotation teams reviewed a total of 103 of the preselected studies with associated sample records and attempted to enrich the contextual information for the samples based on information available on the Web and in the formally published scientific literature. Annotators added contextual information in the form of key-value pairs for sets of sample records and using color-coding logged new, corrected and contradictory information to that originally submitted at the time of data deposition.

**Figure 1:**
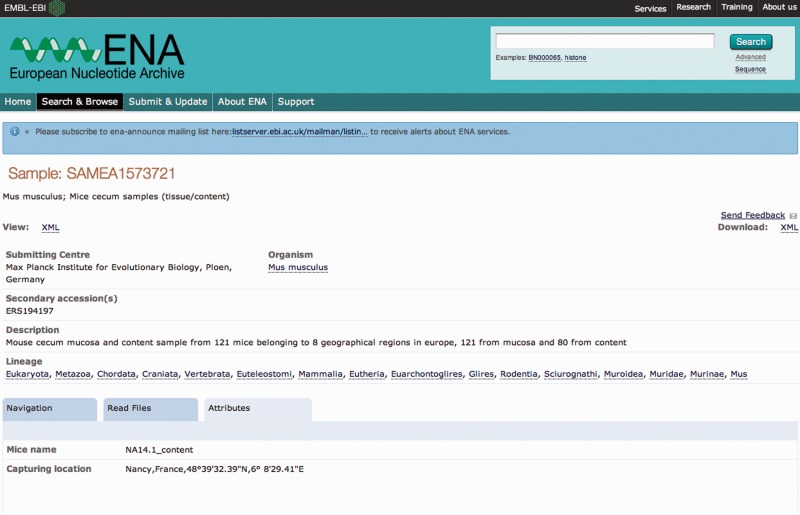

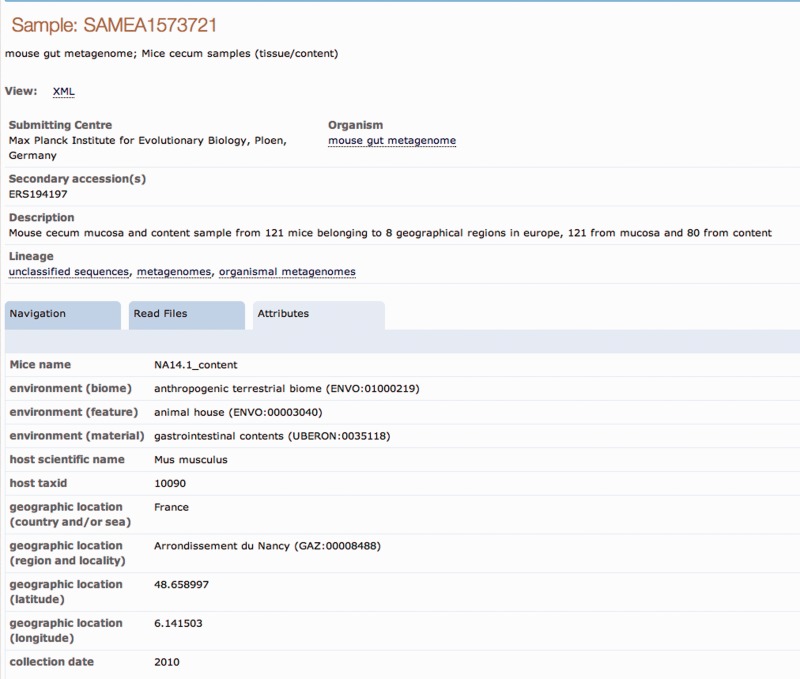
An example of a sample record improvement by the Sample Record Annotation Workshop. The *Attributes* tab of the ENA sample record SAMEA1573721 is shown here with the originally submitted contextual data (**a**) and expanded annotation as a result of the Workshop (**b**).

Comprehensive sequence data records are provided by INSDC partners acting as hosts of the data. As such, ownership, and hence editorial control, remains with the data generating group. In the second stage of the annotation experiment, ENA team members selected from all sample records reviewed during the SRAW those records originally submitted with no or minimal sample contextual data and where thus the added value of SRAW annotation is highest (Figure 1). Owners of these sample records were contacted for their consent to update the records with the SRAW descriptors.

In the majority of the reviewed sequencing studies, it was possible to assign a core set of key-value pairs to all sample records within a study. In addition, there were specific keys where values varied between samples within a study, such as a sampling locality or subject age. During the SRAW, annotators listed sample record accessions, where these specific descriptors should be used and did not create a set of descriptors for each sample record. This minimized editing steps and maximized the number of sample records reviewed during the jamboree.

For each sample, a full set of contextual key-value pairs was created from the study- and sample-level sets and loaded into ENA. This time-consuming step was carried out in the third stage of the annotation exercise and only for sample records of consenting owners.

## Post-deposition exercise results

### Annotator’s comments

The SRAW annotators highlighted several interesting aspects that had an impact on the annotation work:

#### 1. Annotation complexity

Representing some of the more complex information relevant to interpreting a study’s context with simple key-value annotations proved unsupportable during this exercise. To illustrate, the study PRJEB3348 focuses on transplantation of a fecal microbiome from a Malawian twin pair discordant for Kwashiorkor into gnotobiotic mice. These mice were then fed a nutrient poor diet typical of rural Malawi prior to nutritional therapy. Following therapy, the nutrient poor diet was readministered. Annotators considered representing this information as the microbiome’s environment using ENVO classes; however, while elements of this temporally extended environmental succession can be captured with ENVO, a small number of ontology classes expressing this sequence would be over-specified and inappropriate for inclusion in a domain ontology. To represent sufficiently this procedure, a more sophisticated contextual data storage solution, such as a resource description framework (RDF) triple-store, is needed. Annotators can then draw from multiple domain ontologies such as ENVO, the OBO Relations Ontology [RO; (23)], and the Ontology for Biomedical Investigations [OBI; (24)] to grant them the flexibility to express more complex entities.

#### 2. Annotation depth

As a part of the workflow, annotators had a prioritized list of descriptors at hand during the annotation process. This list included ENVO classes and mandatory descriptors from the ENA sample checklists (5) applied to sample records as appropriate. The following descriptors were prioritized: classes from the ‘biome’(ENVO:00000428), ‘environmental feature’ (ENVO:00002297) and ‘environmental material’ (ENVO:00010483) hierarchies within ENVO, geographic location (longitude, latitude, country, locality, depth, elevation, altitude), collection date, collected by, marine sampling information (site, platform campaign, protocol, temperature and salinity), host-related descriptors (scientific name, taxid, subject ID, status, disease, isolation source) and pathogenicity.

The scientific focus of each sequencing study can determine additional relevant descriptors. Often these descriptors are specific for a small subset of samples, such as the host diet in the studies PRJEB1147-PRJEB1152. Annotation with these descriptors places significant time demands when reviewing sample records and, in contrast with broader descriptors, because it offers very esoteric information, provides only limited gains in terms of discoverability.

#### 3. Georeference of clinical metagenomic samples

While costs associated with collection of geolocation information are typically negligible (e.g. through ready availability of capture systems in smartphones), costs of capturing this information in the archive persist due to the resources required for requesting, updating and retrofitting the information. In order to balance these costs and considering relevance of geolocation to clinical samples of patients in hospital or metagenomes of laboratory mice, the archive made a pragmatic decision not to expect descriptors that pertain to geolocation for clinical samples. However, the country and/or geographic locality of the hospital/laboratory are frequently provided and the metadata standard *Minimal Information about Metagenome Sequence* [MIMS, (25)] requires reporting of geolocation coordinates for sequenced metagenomic material. The annotators argued that it is incorrect to infer the coordinates based on the submitted locality information for the purpose of compliance to the MIMS standard. This would suggest a revision of the MIMS for clinical and laboratory metagenomic samples. Adoption of a controlled null value vocabulary (26), which would allow cases to be specified where georeference reporting is not applicable, could highlight scenarios where this issue exists.

#### 4. Relevance of associated literature

Only a small number of the reviewed study records were associated with a reference to formally published scientific literature. However, such references would be extremely useful for validating provenance information submitted to sequence databases against facts published in the scientific literature, Well-referenced records can assist curators in detecting clear errors such as the metagenomic data derived from microbial communities associated with a red deer submitted as a ‘bovine metagenome’ (sample record ERS196168).

### Annotation errors

During the SRAW, annotators revealed several recurrent annotation errors. While easy to portray these as simple data entry errors during deposition, their existence likely reflects a balance of some combination of factors that may include, on the data provider side, low awareness of the importance of accurate reporting, misunderstanding of documentation, pressure to work at speed and, on the data archive side, inaccessible or unclear documentation, a suboptimal interface and insufficient real-time validation and feedback as data are entered.

(1) An essential descriptor distinguishing between treatments of samples (often referred to in transcriptomics as the experimental ‘factor’) is frequently not provided, such as the distinction between lean and obese sample treatments in the study PRJEB4245.

(2) Measurement units are reported as a part of the descriptor *value* rather than separately in the *units* section of the relevant descriptor definition.

(3) Geolocation coordinates are provided in degrees and minutes rather than in expected decimal degrees, as explicitly emphasized in the definition of the georeference descriptors. In other cases, the coordinates are provided but have little use since they lack essential precision. This includes cases where only geolocation degrees are submitted, or the GoogleMap results of the locality where the study took place are reported. For instance, the sample records of the study PRJEB4336 all have the coordinates of Copenhagen since the study took place in Denmark.

(4) Although standardized informative keys are available, depositors frequently and unnecessarily use their own user keys. This results in either these keys not being searchable or the need of maintaining mappings between keys representing the same concept, which is costly to maintain and not comprehensive.

The archive will further facilitate usage of authorized keys, e.g. by allowing a global search of existing keys across all available checklists in the interactive submission tool Webin, as opposed to keys being currently searchable only within the selected checklist. However, our experience shows that depositors frequently do not appreciate the fundamental value of reporting contextual information and as a consequence do not invest an effort in its accurate provision.

(5) The value of using ontology terms is frequently not fully appreciated by depositors since ontology terms are often used without the essential term ID or in an incorrect format, instead of the format expected by the repository <*term (ontology:identifier)>*, for example *anaerobic sludge (ENVO:00002129)*.

### Use of approved ontology classes

Due to the environmental nature of the selected sample records the Environment Ontology (14) was frequently used in the sample annotation process. Annotators used classes from the biome, environmental feature, and environmental material hierarchies to populate the keys *broad ecosystem context* (biome), *local environment determined by* (feature), *surrounded by* (material) and *partially surrounded by* (material). Several new classes were requested and subsequently resolved by ENVO editors via the ENVO issue tracker system (21). These include:polar desert biome (ENVO:01000186), human house (ENVO:01000418), root matter (ENVO:01000349), autoclaved sand (ENVO:01000350), gastrointestinal contents (UBERON:0035118), old plant (ENVO:01000413), young plant (ENVO:01000414), maize field (ENVO:01000348), rhizosphere (ENVO:00005801) and carbon nanotube-enriched soil (ENVO:01000427). The semantics of these classes will be continually improved and interlinked with other ontologies by successive rounds of curation by the ENVO editorial team, increasing the value of these annotations well into the future.

UBERON (15), a cross-species ontology of anatomical structures, was another ontology from which annotators drew classes. Since a number of reviewed sequencing studies focused on clinical samples or laboratory host–microbiome analysis, this ontology allowed an accurate annotation of anatomical structures determining the local environment of sequenced material. Linking UBERON classes to ENVO’s environmental system (ENVO:01000254) class through the ‘determined by’ (ENVO:2100001) relation allows semantically coherent usage of classes across relevant ontologies, tailored to metagenomic record annotation.

Geographic provenance of the sequenced samples was reported using the INSDC country list (4), MarineRegions (27) and GAZ (28), a gazetteer built using ontological principles, for precise specification of localities. In several cases the Experimental Factor Ontology (EFO) (29) was used to capture diseased state of the sampled organism or host.

Scientific names of the sequenced organisms were annotated using the taxonomic index of the NCBI Taxonomy (30).

**Figure 2:**
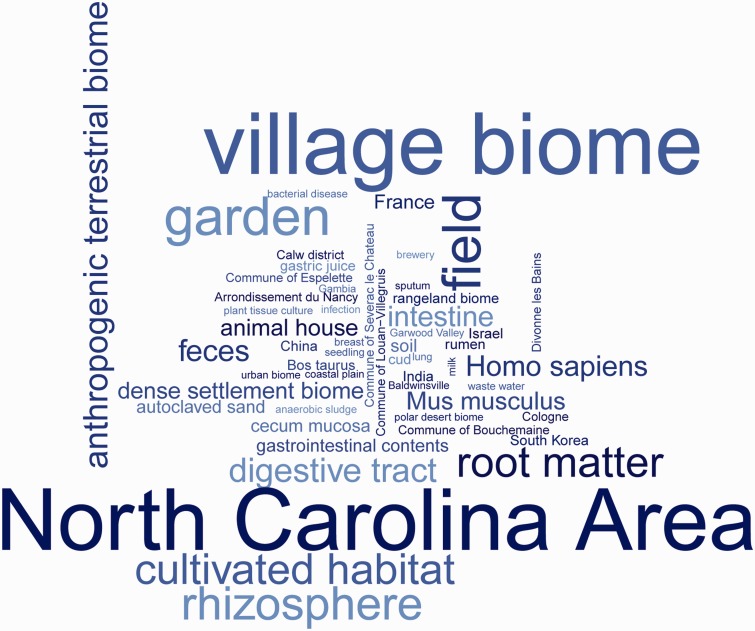
Word cloud of ontology classes annotated in the sample records as a result of the environmental Sample Record Annotation Workshop. The word cloud illustrates frequency of ontology classes usage summarised in Table 1.

Annotators attempted to extend the sample record descriptors as accurately as possible based solely on information available in the public domain. In cases where more specific information was not available, a higher level ontology class was used, rather than selecting a more specific class based on potentially incorrect assumptions. Table 1 summarizes ontology classes used for the annotation of sample records in ENA. Figure 2 depicts a word cloud of the ontology classes used in the annotation of ENA sample records.

**Table 1.** An overview of ontology classes (with their unique class identifiers), the number of ENA sample records annotated with these classes and ENA study accessions associated with the annotated sample records

Ontology class	Ontology class unique ID	Ontology class frequency	ENA study accession
village biome	ENVO:01000246	1277	PRJEB2989
anthropogenic terrestrial biome	ENVO:01000219	395	PRJEB638, PRJEB1391, PRJEB1720, PRJEB7248, PRJEB7112, PRJEB5976
dense settlement biome	ENVO:01000248	207	PRJEB4413, PRJEB4562, PRJEB3374
rangeland biome	ENVO:01000247	48	PRJEB5982
urban biome	ENVO:01000249	7	PRJEB4512
polar desert biome	ENVO:01000186	4	PRJEB3228
garden	ENVO:00000011	723	PRJEB2989
field	ENVO:00000114	723	PRJEB2989
cultivated habitat	ENVO:00000113	550	PRJEB2989
digestive tract	UBERON:0001555	388	PRJEB1391, PRJEB4413, PRJEB1720, PRJEB7112, PRJEB4562, PRJEB3374
intestine	UBERON:0000160	315	PRJEB4413, PRJEB1720, PRJEB7112, PRJEB4562, PRJEB3374
animal house	ENVO:00003040	200	PRJEB638
rumen	UBERON:0007365	48	PRJEB5982
lung	UBERON:0002048	8	PRJEB7248
infection	EFO:0000544	8	PRJEB7248
bacterial disease	EFO:0000771	8	PRJEB7248
brewery	ENVO:00003885	7	PRJEB4512
anaerobic sludge	ENVO:00002129	7	PRJEB4512
breast	UBERON:0000310	6	PRJEB5976
coastal plain	ENVO:00000090	4	PRJEB3228
plant tissue culture	ENVO:02000009	4	PRJEB2989
seedling	TAIR:0000027	4	PRJEB2989
rhizosphere	ENVO:00005801	621	PRJEB2989
root matter	ENVO:01000349	536	PRJEB2989
feces	UBERON:0001988	332	PRJEB1391, PRJEB4413, PRJEB7112, PRJEB4562, PRJEB3374
gastrointestinal contents	UBERON:0035118	136	PRJEB638, PRJEB1720
soil	ENVO:00001998	124	PRJEB3228, PRJEB2989
cecum mucosa	UBERON:0000314	120	PRJEB638
autoclaved sand	ENVO:01000350	120	PRJEB2989
cud	UBERON:0012114	48	PRJEB5982
gastric juice	UBERON:0001971	48	PRJEB5982
sputum	UBERON:0007311	8	PRJEB7248
waste water	ENVO:00002001	7	PRJEB4512
milk	UBERON:0001913	6	PRJEB5976
North Carolina Area	GAZ:00082924	1277	PRJEB2989
France	GAZ:00002940	147	PRJEB4413
South Korea	GAZ:00002802	73	PRJEB1391
China	GAZ:00002845	60	PRJEB1720, PRJEB3374
Israel	GAZ:00002476	52	PRJEB7112
India	GAZ:00002840	48	PRJEB5982
Commune of Espelette	GAZ:00321111	36	PRJEB638
Commune of Severac le Chateau	GAZ:00372953	26	PRJEB638
Commune of Bouchemaine	GAZ:00377283	25	PRJEB638
Commune of Louan-Villegruis	GAZ:00365581	24	PRJEB638
Calw district	GAZ:00020488	24	PRJEB638
Arrondissement du Nancy	GAZ:00008488	22	PRJEB638
Divonne les Bains	GAZ:00059221	22	PRJEB638
Cologne	GAZ:00396037	21	PRJEB638
Gambia	GAZ:00000907	8	PRJEB7248
Baldwinsville	GAZ:00223041	7	PRJEB4512
Garwood Valley	GAZ:00139908	4	PRJEB3228
Homo sapiens	NCBI:9606	293	PRJEB4413, PRJEB7112, PRJEB4562, PRJEB1720, PRJEB7248, PRJEB5976
Mus musculus	NCBI:10090	236	PRJEB638, PRJEB7112, PRJEB3374
Bos taurus	NCBI:9913	48	PRJEB5982

The NCBI Taxonomy hierarchy has been included here for an overview of the records taxonomic coverage.

Annotators focused on the addition of authorized sample descriptors from data standards supported by the ENA, such as GMI (31) or MIxS (32). Where possible, descriptors such as collection date, host information and geo- location, expressed in terms of political regions or coordinates, were annotated. However, reaching compliance to a particular molecular data standard was not feasible here due to very minimal information available for same sample sets. Furthermore, the molecular data standard MIxS, developed by the Genomic Standards Consortium (33), currently supports only MIxS ENVO entries. UBERON classes have to be imported into the ENVO or the correct semantics has to be asserted in a triplestore. The annotation exercise highlighted the need for users to annotate environmental features using classes from a range of domain ontologies and appropriately linked to the semantics represented in ENVO.

## Conclusions

The environmental Sample Record Annotation Workshop reviewed 103 ENA studies. 13 studies with minimal or no sample annotation, Table 2, were selected and consent for the update of their associated sample records requested. Subsequently, individual sample records were enriched with annotations resulting from the workshop. In total 1939 sample records were updated and became eligible for data analysis by EMG and other resources. At the start of the SRAW, EMG contained 1750 public sample records associated with sequence data and metagenomic analyses. The annotation of 1939 sample records has more than doubled the number of samples eligible for the metagenomic analysis. Primary sequence data from 1688 of these samples passed the EMG read data quality control. These metagenomes were analysed and are now discoverable and available (34) via the EMG portal.

**Table 2.** A list of ENA studies and the number of associated sample records updated with annotation results of the Sample Record Annotation Workshop

ENA study	Number of sample records
PRJEB2989	1277
PRJEB638	201
PRJEB4413	147
PRJEB1391	73
PRJEB1720	56
PRJEB4562	56
PRJEB7112	52
PRJEB5982	48
PRJEB7248	8
PRJEB4512	7
PRJEB5976	6
PRJEB3228	4
PRJEB3374	4

Although impactful in its domain, clearly, as expected, our approach was not perceived to be a scalable solution. The preparation phase of the SRAW required 160 person hours, covering all correspondence with external annotators, logistics for visiting scientists and tutors, master annotation file preparation, exploration of relevant ontologies and annotation environments.

The SRAW itself required 480 person hours, including the time of 13 annotators working 7 h per day for 5 days, extended with 30 h logistics and tutor’s time. One could argue that training annotators would reduce the person hours here. However, this will not affect the postdeposition curation costs since the archive is not in the position of establishing a permanent team of ontologists and costs of training also need to be covered.

The post jamboree phase required 150 h and included review of the annotation results, contacting submitters, updating records in the database, and resolving ENVO tracker issues. Any follow-up data analysis of updated samples by EMG is not included in this calculation.

In total, the sample Record Annotation Workshop required 794 person hours leading to direct improvement of contextual information in 1939 environmental sample records.

Based on this, we derive an annotation rate of one sample per 0.4 h per annotator. It would take one working week to annotate a fairly standard dataset of 100 samples. A team of 37 full time staff would therefore be needed to handle all samples submitted through ENA at the current rate to the depth achieved during this annotation exercise. Moreover, annotators, as third parties, are limited to information available to them only the public domain. In contrast, submitters familiar with their own contextual information, when provided via user-oriented reporting systems, such as those supporting submissions of environmental data—Webin (35) or Metazen (36)—would need a fraction of this time accurately to report minimal contextual data.

Although repositories continue to improve tooling, user instructions and training materials to facilitate reporting, we believe that an understanding of the fundamental importance of contextual data is essential in driving up the quality and richness of reporting. Primary data archives will continue to demonstrate to depositors the value of contextual data by improving data discovery services and by engagement in outreach activities directly or in liaison with other resources, which can add value to primary data only if sufficient contextual data were deposited into the primary data archive.

While the annotation exercise proved to be a time- and resource-demanding effort, we have benefited from it in a number of ways:

1. We have confirmed that the application of classes from an ontology is a process that requires concurrent development of the ontology itself. In addition, we confirmed that a satisfactory description of an environmental sample requires classes from multiple domain ontologies, interlinked through appropriate semantics.
2. We discovered that an efficient workflow for this kind of annotation is application of ontology classes across a group of sample records rather than the more obvious workflow of studying one sample record at a time and applying classes from multiple ontologies. This knowledge may be useful for instance in designing rule-based systems to scale annotation work.
3. The Workshop gave the ENA curation team a working knowledge of the ontologies addressed that will need to be implemented in, and supported for, any submission system where data submitters or other users are asked to provide annotation from such ontologies.
4. The Workshop provided a well annotated data set that will (i) serve as an example to submitters and consumers of how annotation should be applied, (ii) serve as use cases for developing discovery and analysis services and (iii) demonstrate to stakeholders the value of standardized annotation.
5. We concluded that such annotation activity, which brings curators, domain scientists and ontologists together to look at real data sets and annotation practices, is a prerequisite for the implementation of any submissions or data presentation services around the ontologies in question. During such an activity, the challenges faced by each group of stakeholders can be shared and solutions discussed and implemented. Such interaction will foster more practical and integrated developments across each area of expertise.
6. Common dogma, that ownership of data records by a data provider prevents improvement of the records by a third party, is not in line with our experience here. We received no in-principle disapproval when asking submitters consent to update their records. Particularly, in cases where very limited annotation existed prior to the Workshop, there were no objections to updating the existing records.

The third party annotation work, described here, highlighted the significant impact of annotation on discoverability and downstream use of annotated data and the benefit that such effort can bring to future data archiving operations. However, it also emphasized its significant costs and the need for a submitter-driven annotation system as a sustainable curation solution.

## Funding

European Molecular Biology Laboratory (EMBL); UK Biotechnology and Biological Sciences Research Council under Metagenomics Portal [BB/I02612X/1].

Funding for open access charges: European Molecular Biology Laboratory (EMBL).

*Conflict of interest*: None declared.
